# 肺原位腺癌的诊断与治疗进展

**DOI:** 10.3779/j.issn.1009-3419.2017.09.09

**Published:** 2017-09-20

**Affiliations:** 200039 上海，上海交通大学附属胸科医院/上海市肺部肿瘤临床医学中心 Shanghai Chest Hospital, Shanghai Jiaotong University, Department of Thoracic Surgery, Shanghai Lung Tumor Clinical Medical Center, Shanghai 200039, China

**Keywords:** 肺原位腺癌, 病理特征, 诊断, 治疗, Adenocarcinoma *in situ*, Pathological characteristics, Diagnosis, Treatment

## Abstract

肺原位腺癌（adenocarcinoma *in situ*, AIS）于2011年在肺腺癌国际多学科分类中新增，2015版世界卫生组织（World Health Organization, WHO）肺肿瘤分类中补充，由于AIS处于肺腺癌发展的早期阶段，加深对其病理、诊断、鉴别与治疗策略等方面的认识，对改善肺腺癌的治疗预后具有重要意义。本综述将对目前AIS的发生发展、病理特征、鉴别诊断和治疗策略等方面的研究做一系统回顾，旨在为AIS的进一步研究提供理论基础。

## Abstract (en)

Adenocarcinoma *in situ* (AIS) is a new concept which was introduced to the 2011 The International Association for the Study of Cancer (IASLC)/ American Thoracic Society (ATS)/ European Respiratory Society (ERS) International Multidisciplinary Classification of Lung Adenocarcinoma firstly and an important supplement of The 2015 World Health Organization Classification of Lung Tumors. Because AIS is at an early stage of development of lung adenocarcinoma, the deepening understanding of its pathology, differential diagnosis, treatment strategies, has an important significance for the improvement of the prognosis of lung adenocarcinoma. This review will provide a systematic review of the main progress of occurrence and development, pathological characteristics, differential diagnosis and treatment strategy of AIS, in order to provide theoretical basis for the further research of AIS.

2011年2月，国际肺癌研究学会（The International Association for the Study of Cancer, IASLC）、美国胸科学会（American Thoracic Society, ATS）和欧洲呼吸协会（European Respiratory Society, ERS）联合发表了关于肺腺癌的国际多学科分类^[1]^。该分类由临床、放射、分子生物学及病理等多学科的专家参与制定。与之前的世界卫生组织（World Health Organization, WHO）（2004年）肺腺癌分类相比，删除了肺腺癌分类中关于细支气管肺泡癌（bronchioloalveolar carcinoma, BAC）的内容，根据近年来的流行病学研究及临床实践，新增关于原位腺癌（adenocarcinoma *in situ*, AIS）、微浸润性腺癌（minimally invasive adenocarcinoma, MIA）等内容。其后，2015版WHO肺肿瘤分类^[2]^关于腺癌的分类及诊断标准也几乎完全延续了2011肺腺癌国际多学科分类中的相关内容。肺原位腺癌由于处于肺腺癌发生发展的早期阶段，其发展演变的机制、病理形态、诊断鉴别、治疗和预后都有一定特殊性，故加深对肺原位腺癌的理解有重要的价值。

## 1 AIS的演变机制

对于AIS的发展，目前主要存在两种观点：一种是线性多步骤发展模式，即AIS是由先兆病变，如不典型腺瘤样增生（atypical adenomatous hyperplasia, AAH）发展而来，随后逐渐发展为附壁样生长为主型的非黏液性腺癌（lepidic predominant invasive adenocarcinoma, nonmucious, LPA）等浸润性腺癌（invasive adenocarcinoma, IA），AIS是肺腺癌的一个阶段形态。Yatabe等^[3]^支持这一观点，他们发现在腺癌组织中，无论是位于中心的浸润灶还是周围的附壁生长灶，癌细胞中都存在*EGFR*基因突变，但只有中心浸润灶发生了*EGFR*基因扩增。另一种观点则认为肺腺癌只是肿瘤细胞沿着不同的分子演变途径发展而来的肿瘤细胞的一个存在形式，不同的肿瘤细胞其可演变的程度是不同的。按照前一种观点，随着肿瘤的发展，肿瘤组织中发生*EGFR*及*KRAS*等基因改变的细胞数目应当是逐渐增加的，但事实上*EGFR*、*KRAS*基因的突变并未按比例发生改变。另外，通过对*AIS*基因杂交的分析，发现一些AIS并没有能力发展为浸润性腺癌。此外，不少临床研究也证实，一部分在影像学上表现为磨玻璃形态的小结节，不但在结节大小等特征上并未随着时间发生改变，而且这类患者与未携带小结节的患者相比，在生存预后上并未存在明显差异^[4]^。尽管有相关研究试图探索AIS的进展和一些特定的基因突变之间的联系，也有一些研究发现*EGFR*基因突变与LPA^[5]^、*KRAS*基因突变与黏液型腺癌^[6]^等存在联系，但目前认为^[7]^，在肺腺癌的各个亚型与相关启动基因之间不存在特定的组织分子联系。

## 2 AIS的诊断和鉴别

在WHO（2004）肺腺癌分类中，对BAC的定义为肿瘤细胞沿着肺泡壁呈附壁样生长，无血管、淋巴组织、胸膜浸润。除了BAC，其他类型的肺腺癌均不主要呈附壁样生长^[8]^。但在临床实际中，呈附壁样生长的腺癌，既有未浸润，也有已经浸润血管、淋巴组织、胸膜者。在不同的浸润情况下，肺腺癌的预后情况并不尽一致，相应的治疗方式也不同。正因为如此，2011肺腺癌国际多学科分类将BAC的概念分散于AIS、MIA和LPA之中^[1]^。

### 2.1 AIS的病理和影像

在2011肺腺癌国际多学科分类和2015版WHO肺肿瘤分类中，AIS的组织学表现为：局限化病变，直径≤3 cm，肿瘤细胞完全沿肺泡壁生长，无间质、血管或胸膜浸润，没有形成乳头或微乳头状结构，肺泡无肿瘤细胞侵及。亚分类包括非黏液性（nonmucinous）和黏液性（mucinous），其中以非黏液性最常见，主要由肺泡Ⅱ型上皮细胞和（或）Clara细胞构成。黏液型AIS则由高柱状细胞构成。无论是非黏液型AIS还是黏液型AIS，都没有（或者说至少没有明显的）细胞核异型性。非黏液性AIS在CT上主要表现为纯磨玻璃结节，黏液型AIS则表现为部分实性或实性结节。AIS的纯磨玻璃结节的密度值通常比AAH要高一点。AIS可以呈现单发或多发^[1, 2]^。

### 2.2 AIS的鉴别

AAH为沿着肺泡壁或者小气道轻度或中度增生的组织团块，由不规则排列的肺泡Ⅱ型上皮细胞和（或）Clara细胞构成，呈局限性生长，团块直径≤5 mm。细胞间隙通常可见，由圆形、立方形、低柱状细胞或者由呈圆形或椭圆型细胞核的peg细胞构成。细胞核活动频繁。从AAH发展到AIS包含有一系列的细胞形态学变化。从组织细胞学上对直径≤5 mm的AAH和AIS进行鉴别很难，或几无可能。AAH在CT上表现为≤5 mm的纯磨玻璃结节，可为单发或多发^[1, 2]^。

MIA为肿瘤直径≤3 cm的孤立性腺癌，主要呈附壁样生长，最大浸润深度≤5 mm，包括侵及肌纤维母细胞的基质在内的浸润部分，形态区别于任何一种浸润性腺癌（腺泡型、乳头型、微乳头型、实性结节型、胶体型、肠型、胎儿型以及黏液性浸润性腺癌），且需要排除淋巴道转移、血道转移、呼吸道转移、胸膜转移和肿瘤坏死。亚型上，大多数MIA为非黏液性，少数为黏液性，主要由肺泡Ⅱ型上皮细胞和（或）Clara细胞构成。准确区分AIS和MIA主要依赖于对完整肿瘤组织标本所进行的病理分析，如果条件允许，则还可以进行分子水平的诊断。仅有一块活组织或冰冻样本，不足以区别AIS和MIA。在CT上，非黏液型MIA主要表现为以磨玻璃成分为主，中心实性成分≤5 mm的部分实性结节。黏液型MIA则表现为实性或部分实性结节^[1, 2]^。通过对2011肺腺癌国际多学科分类和2015版WHO肺肿瘤分类的解读，不难发现AAH、AIS和MIA在影像学表现上有部分重叠。

LPA沿着肺泡壁表面生长，其细胞形态和AIS、MIA接近，由肺泡Ⅱ型上皮细胞和（或）Clara细胞构成，组织学表现以附壁样生长为主（非腺泡性、乳头性、微乳头性和实性），至少有一处病灶浸润直径大于5 mm，已经浸润至淋巴组织、血管、胸膜，或者出现肿瘤坏死。在CT表现上，LPA通常为部分实性结节，其磨玻璃成分与实性成分各自所占比例不固定^[9]^。Jane等^[10]^比较AIS/MIA与LPA在CT上实性成分所占体积百分比与病理类型的关系，发现对于部分实性结节，AIS/MIA为8.2%（95%CI: 2.7%-13.7%），LPA为14.5%（95%CI: 10.3%-18.7%）。

### 2.3 AIS的临床诊断

鉴于AIS与IA在治疗方式和长期预后等方面存在较大的不同，而两者在CT影像表现上有时较难区分，故不少研究者都进行了这一方向的研究。Lim等^[11]^的一项旨在探讨直径≥10 mm的纯磨玻璃结节的病理类型与预后的研究发现：支气管充气征、结节直径和结节重量是区分AIS和IA的影响因素。Miyata等^[12]^研究了早期黏液性腺癌（指AIS和MIA）在高分辨CT下的表现，发现所有的早期黏液性腺癌都表现为实性或部分实性结节，并且其中的大部分结节都有含气腔；而相比于正常软组织，早期黏液性腺癌在CT上的衰减系数更小。早期黏液性腺癌因中央纤维成分的降解而导致肿瘤边界收缩，故常出现胸膜凹陷。一部分早期黏液性腺癌在CT表现上与炎症类似，不易区分。在术后病理分期方面，Lim等^[11]^建议在T分期上以浸润部分直径，而非包含附壁样生长成分来计算肿瘤直径；在多发结节的M分期上，应先检查各个结节的组织学亚型与分子特征后，判断是否为多原发还是肺内转移灶。国内学者在探讨高分辨CT下AIS/MIA与IA的表现差异时发现：结节直径≥12.2 mm，实性成分≥6.7 mm，实性部分CT值≥-192 HU，且含有支气管充气征的磨玻璃结节为IA可能性大^[13]^。Yanagawa等^[14]^通过对薄层CT下不同恶性程度腺癌的观察，发现与MIA或IA相比，AIS的特征为：边界规则，未见伴有中断或不规则扩大的支气管充气征，胸膜凹陷少见，实性部分大小 < 5.3 mm。Si等^[15]^在观察纯磨玻璃结节中AAH、AIS和MIA各自特征时发现：AIS的纯磨玻璃结节大小≤7.5 mm，且毛刺及胸膜凹陷征少见。

正电子发射计算机断层成像（positron emission tomography, PET/CT），通过测量氟脱氧葡萄糖的标准摄取值（standard uptake values, SUVs）来反映原发肿瘤的细胞增生和侵袭性，在肿瘤分期、结节直径≥7 mm的浸润型腺癌患者的随访观察、浸润性腺癌患者化疗后的疗效评估等方面有较大优势。近年关于PET/CT和AIS诊断相关的研究较多^[16-18]^，但PET/CT对AIS的敏感性尚不够理想，在AIS的诊断方面优势不大^[1, 2]^。另外，早期肺腺癌（AIS和MIA）的组织异质性不高，故不推荐对AIS进行术前组织细胞活检^[1]^。

## 3 AIS的治疗

自2011肺腺癌国际多学科分类引入AIS后，对于AIS的治疗，相关研究已经证实在实现完整切除后，AIS可以实现100%的5年无病生存期，且不需要行术后辅助性放疗或化疗。但相关手术问题依然存在争议，有待进一步探讨。

### 3.1 切除范围

对于亚肺叶切除能否实现早期肺腺癌（AIS和MIA）的充分治疗，单中心的回顾性研究^[1]^提示对于肿瘤大小≤2 cm的早期肺腺癌，亚肺叶切除和标准的肺叶切除在生存预后上并无显著差异，但也有研究^[19]^发现一部分磨玻璃结节患者在行局部切除后出现了切端（缘）复发。由于缺乏相关随机对照试验（如正在进行的日本的JCOG0802、JCOG0804和美国的CALGB140503）的研究支持，目前尚不能确认亚肺叶切除能够像肺叶切除一样实现对早期肺腺癌的充分治疗^[1, 2, 7]^。

### 3.2 术前高分辨薄层CT的作用

有关研究已经证实术前高分辨薄层CT的影像表现与术后病理类型存在相关关系^[20]^，而病理类型对手术方式的选择具有重要意义。但如何在术前借助高分辨薄层CT挑选适合亚肺叶切除的患者，如病灶位于周围或中央，表现为磨玻璃样结节或实性结节，肿瘤大小在T1a、T1b还是T1c等方面^[1]^，目前都存在争议，亟待相关研究予以完善。

### 3.3 淋巴结清扫

是否需要对早期肺腺癌行系统性淋巴结清扫？有研究^[21]^通过对821例AIS和6, 137例MIA患者进行分析后发现，超过99.9%的AIS和MIA并未出现淋巴血管侵犯，且相关的多中心前瞻性随机临床试验已经证明：对于T1-2N0的非小细胞肺癌患者，系统性淋巴结清扫和采样相比，在长期生存上并无优势^[1]^。故对于早期肺腺癌，并不推荐行系统性淋巴结清扫。

### 3.4 术中冰冻病理

由于术前无法明确病灶病理类型，术中冰冻对于判断局部切除（包括解剖性亚肺叶切除和楔形切除）的距离是否充分、肿瘤是否有胸膜和淋巴血管侵犯等具有非常重要的价值。有关研究者认为术中冰冻活检的特异性较高，但敏感性较差，较常出现的一个问题是容易高估肿瘤的浸润程度^[22]^。

## 4 小结与展望

在我国，肺癌是最常见且致死率最高的恶性肿瘤，其中又以肺腺癌比例最高^[23]^。作为肺腺癌发展的早期阶段，AIS的概念自2011肺腺癌国际多学科分类提出以来，很快就得到了国内国外研究肺癌的专家学者的认可与重视。鉴于AIS在发展到LPA等浸润性腺癌后，患者的生存期会出现明显下降，因而对AIS的早发现、早诊断、早治疗具有重要的临床意义。但由于目前临床上关于AIS的手术指证和手术方式结论多源于单中心的回顾性临床研究，缺乏多中心随机对照及相应的基础研究支持，因此在国内外始终没有形成一个比较公认的诊疗指南。基础研究方面，有关学者已经发现在AIS演变至LPA的过程中，肿瘤细胞微环境会发生改变^[24]^，结合对肺腺癌非线性多步骤发展模式的探索，期待进一步深入细致的临床——基础研究揭示更多AIS的奥秘。
